# “The Day He Fell Ill, We Turned on a Switch…Now, Everything Is My Responsibility”: Scoping Review of Qualitative Studies Among Partners of Patients with Cancer

**DOI:** 10.3390/curroncol33020069

**Published:** 2026-01-24

**Authors:** Preet Kang, Ursula Ellis, Jacquelyn J. Cragg, A. Fuchsia Howard, Amirrtha Srikanthan, Niki Oveisi, Mary A. De Vera

**Affiliations:** 1 Faculty of Pharmaceutical Sciences, University of British Columbia, Vancouver, BC V6T 1Z4, Canada; preetkrkang@gmail.com (P.K.);; 2 Collaboration for Outcomes Research and Evaluation, Vancouver, BC V6T 1Z4, Canada; 3 Woodward Library, University of British Columbia, Vancouver, BC V6T 1Z4, Canada; 4 Faculty of Applied Science, School of Nursing, University of British Columbia, Vancouver, BC V6T 1Z4, Canada; 5 Faculty of Medicine, University of Ottawa, Ottawa, ON K1N 6N5, Canada; 6 Department of Medicine, Division of Medical Oncology, The Ottawa Hospital, Ottawa, ON K1N 6N5, Canada; 7 The Ottawa Hospital Research Institute, Ottawa, ON K1N 6N5, Canada; 8 Centre for Advancing Health Outcomes, Vancouver, BC V6T 1Z4, Canada

**Keywords:** cancer patient, partner, qualitative research, lived experiences, scoping review

## Abstract

Our objective was to conduct a scoping review and narrative synthesis of qualitative studies that examined experiences of partners of cancer patients. We searched MEDLINE, Embase, CINAHL, PsycINFO, and Scopus for qualitative studies involving adult (≥18 years) partners (e.g., in a romantic relationship) of patients diagnosed with cancer and published in English. We extracted findings from included studies, along with key study and participant characteristics, and applied a narrative summary approach, a process that allowed us to identify synthesized themes across studies. Our search identified 15,729 records, of which 159 met the inclusion criteria. Included studies primarily collected data through interviews, with participants being predominantly female and middle-aged (55.2 ± 8.3 years). Partners were commonly in relationships with patients diagnosed with breast, genital–urinary, or gastrointestinal cancer. Our synthesis identified four conceptual themes—transformation of relationship dynamics and roles, distress and burden, coping strategies, and unmet needs and support gaps—which reflect the emotional, relational, and practical challenges partners navigate throughout the cancer trajectory. These findings highlight the need to better recognize and support the role of partners within the cancer care landscape as their wellbeing impacts care and experiences of patients with cancer.

## Simple Summary

Cancer is a complex diagnosis that affects patients and their partners, who often assume the role of a primary caregiver. Partners are confronted with emotional, interpersonal, and practical challenges throughout the illness trajectory. Our review reported that cancer changes relationships due to roles being reshaped. This often leads to stress and the caregiving burden requiring various coping strategies from partners. As survivorship care centers on the patient, the partner experiences unmet needs and gaps in support.

## 1. Introduction

As survival rates continue to rise across most cancer diagnoses, the patient experience is shifting from an acute health condition to a long-term concern [1,2]. Given that survivorship care requires significant psychosocial and financial investment, its strains extend to the community supporting the patient [3,4]. Indeed, cancer affects not only the patient, but those around them, and for those who are coupled, coping with the illness becomes a mutual shared effort [5]. Within this shared context, the presence of a social network, especially the involvement of a partner, has been shown to increase the likelihood that patients with cancer pursue definitive therapy [6], thereby prolonging cancer patients’ survival [7]. However, the burden of caring for the patient—as well as the resulting financial and relationship impacts—can affect partners considerably, especially given that they often assume the primary caregiving roles [4,8,9]. Partners in this study are defined as anyone in an intimate relationship (e.g., spouse, boyfriend, girlfriend, common-law partner) with the patient. Thus, a deeper understanding of partners’ lived experiences is necessary to inform survivorship care and support for improved health outcomes for both partners and patients.

Quantitative research has shown that informal caregivers of patients with cancer, including partners, experience compromised quality of life and health behaviours [10], increased stress levels, and a lack of social support [11]. Quantitative data on this topic have been synthesized to help us understand the breadth of such literature. For instance, Lambert (2012) [12] synthesized 29 studies that assessed the unmet needs of partners and caregivers. This review pooled findings from the manuscripts and compiled the prevalence of unmet needs. The most common unmet needs were related to managing emotional or psychological distress [12]. Ochoa’s (2020) [13] systematic review of 60 quantitative studies on caregivers of cancer patients suggests that caregiver quality of life is shaped by multiple factors (e.g., time spent caregiving, gender, age, relationship to the care recipient, etc.). Prolonged caregiving led to increased caregiver stress and decreased overall quality of life [13].

Qualitative research provides essential context for understanding partners’ lived experiences, offering insight into the multifaceted ways they are affected—well beyond caregiving alone—in ways that quantitative studies cannot fully capture. Anderson (2019) [14] found that caregivers’ unmet needs come from patients’ unmet needs. The emotional tie between the patient and the carer led to the caregiver’s distress [14]. Banks (2023) found that caregivers’ sense of responsibility for patients’ wellbeing stemmed from factors such as fear of the patient suffering, a need to reduce or protect the patient from cancer-related distress, and caregivers’ own experience of uncertainty and unpreparedness [15]. Ussher’s (2011) qualitative analysis traced how the changed relationship led to a duality of feelings for the caregiver—sadness and frustration, as well as feelings of love [16]. While individual studies provide valuable insights into partner caregiving, often focused on specific cancer types, the overall breadth, scope, nature, and collective findings of the qualitative research remain unclear.

To our knowledge, there is no synthesis or evidence mapping of the lived experiences of partners of patients with cancer, despite the plethora of qualitative research on this topic. Because qualitative literature spans many cancer types, each with its own context and nuance, it can be challenging to interpret partners’ experiences in a unified way. A synthesis can illuminate the common threads that cut across diagnoses, clarifying areas of convergence in partners’ experiences. It can also identify gaps that should be explored and prioritized in future research, supporting the development of a more cohesive understanding of partners’ needs. Accordingly, our objective was to conduct a scoping review to explore and map the lived experiences of partners of cancer patients based on qualitative studies.

## 2. Methods

### 2.1. Overview

The research question for this review asks, “What are the lived experiences of partners of cancer patients”? Recognizing that the concept of lived experience encompasses a wide range of personal, emotional, and social dimensions [17], we conducted a scoping review to systematically identify, map, and synthesize existing evidence on how partners experience and respond to a cancer diagnosis. Guided by the Arksey and O’Malley framework [18], our scoping review involved (1) formulating the research question, (2) identifying relevant studies, (3) including studies that met our inclusion criteria, (4) extracting data, and (5) categorizing and presenting the findings. We also followed the PRISMA-ScR (Preferred Reporting Items for Systematic reviews and Meta-Analyses extension for Scoping Reviews) checklist for reporting [19,20]. The review was registered on the OSF database (pre-registration link: https://doi.org/10.17605/OSF.IO/9BMTW; pre-registration date: 8 January 2026).

Guided by the population–concept–context framework by the Joanna Briggs Institute for Scoping Reviews [19], we were interested in original, peer-reviewed qualitative research studies (context) that explored the lived experiences (concept) of partners of patients diagnosed with cancer. For our purposes, we defined “partner” as an individual in a romantic intimate relationship with the cancer patient, regardless of marital status, gender, or cohabitation [21].

### 2.2. Search Strategy and Inclusion Criteria

The search strategy was developed collaboratively with a research librarian (UE) who searched five electronic databases: Embase (Ovid), MEDLINE (Ovid), CINAHL (EBSCO), PsycINFO (EBSCO), and Scopus (Elsevier). Searches were performed from inception to 19 February 2025 (Tables S1–S5 provide the search strategies applied for all databases). Search results were deduplicated using Covidence software (Accessed on 8 January 2025) [22]. Eligible peer-reviewed studies met the following inclusion criteria: (1) used qualitative methods for data collection (e.g., interviews, focus groups) and analysis (e.g., content, thematic, or narrative analysis); (2) explored lived experiences; (3) included samples consisting of partners (e.g., spouse, common law, boyfriend, girlfriend, etc.) of patients diagnosed with cancer or mixed samples (e.g., caregivers, partners) in which partners comprised at least half of participants; and (4) were published in English. We excluded studies that used mixed methods, intervention designs, or survey-based data collection, as well as those that included mixed samples with patients as participants (e.g., dyads), since their inclusion would limit the ability to isolate partners’ experiences. Reviews, commentaries, and opinion pieces were also excluded. Upon extraction (UE), all identified studies underwent initial title and abstract screening, followed by collaborative full-text review to assess eligibility for final inclusion (PK, MADV). Uncertainties were addressed through discussion and consensus amongst reviewers (PK, MADV).

### 2.3. Data Extraction and Synthesis

We extracted information on study characteristics, including publication year, country, study period, study aim, and data collection and analysis methods. With respect to partners’ characteristics, we extracted information on age; sex/gender; number of participants; and, where possible, the nature of the relationship to the patient and terminology used (e.g., partner, spouse, caregiver, etc.). Sex, assigned at birth, is often determined based on biological attributes (e.g., physical and physiological features); meanwhile, gender is a social identity that is determined by one’s internal sense of self [23]. Of note, for studies with mixed samples, we also extracted the proportion of partners in the total sample. When reported, we also extracted information on patient characteristics, particularly cancer type. We also extracted findings from included studies, specifically the results section, including reported themes and quotes. Following recommendations by a guide for narrative synthesis for reviews from Popay et al. (2006), we used a narrative summary approach, which is suited to synthesizing findings from a large body of qualitative research encompassing diverse contexts and participant experiences [24,25]. The text results for each study, despite different methodologies, were used to identify patterns and to develop a summary that contextualized findings. Each study summary was then compared with those of the other studies to identify similarities and differences. These summaries were then organized to develop higher-level synthesized themes that informed the findings of this review. To maintain the integrity of the emerging themes, we repeatedly compared the themes to the study summaries and extracted results sections while maintaining cross-validation between researchers. This process enabled us to integrate evidence across studies while preserving the richness, context, and nuance of partners’ lived experiences. NVivo^TM^, version 14 (QSR International), was used to support the organization and synthesis of qualitative data [26].

## 3. Results

### 3.1. Included Studies

**Figure 1:**
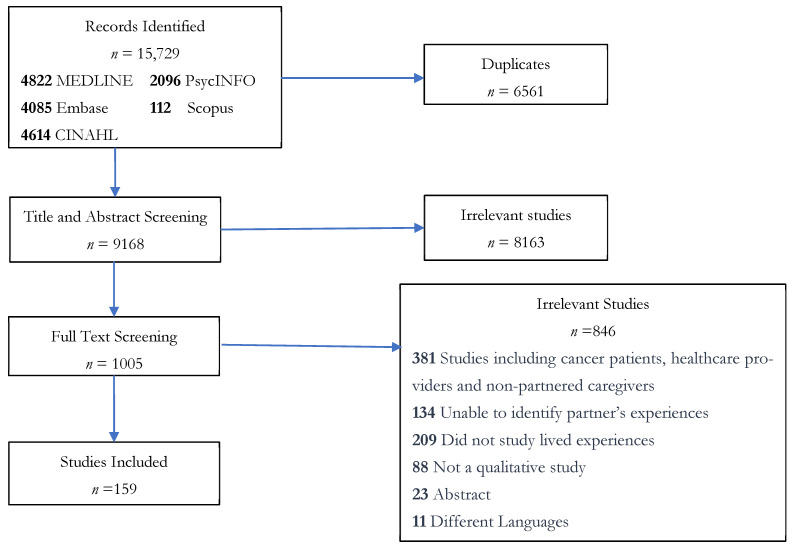
PRISMA diagram of study inclusion.

The initial search identified 15,729 studies, of which 6561 were duplicates, leaving 9168 for title and abstract screening. Upon title and abstract screening, 8163 were further excluded as irrelevant, and 1005 full-text articles were assessed for eligibility. Of these, 159 met the inclusion criteria and were retained for the review, with the corresponding PRISMA diagram shown in Figure 1 (for a full list of included studies, please refer to Table S6).

Table 1 summarizes the characteristics of the included studies. The most common method of data collection was individual interviews, while some studies used both focus groups and personal interviews. Thematic analysis (27%) was the primary analytical framework used in the included studies, followed by content analysis (20.1%) and grounded theory (15.1%). With an increased number of publications starting in 2006, most studies on the topic were published between 2021 and 2025. The majority of the studies were published in Europe (30.8%) and North America (27.7%).

### 3.2. Partner Participants

Table 2 summarizes the characteristics of the study participants in the included studies. The total number of partner participants in the included studies was 3042. Amongst the total sample, 2269 (74.6%) were partners. The reporting of sex and gender was often conflated (e.g., studies used the terms “male/female” and “man/woman” interchangeably without clarifying whether sex or gender was measured or reported). Given the inconsistent reporting of sex and gender across studies, we elected to report participant characteristics as sex, reflecting the terminology most frequently used. A total of 1229 (41%) of participants were recorded as male and 1780 (58%) as female. Most commonly, participants/partners were referred to as carers or caregivers. Participants’ ages ranged from 18 to 80+, with a mean of 55.24 years.

Although not a characteristic of the partners themselves, we also recorded the type of cancer the patients were diagnosed with, as this was relevant to understanding the context of partners’ experiences. Among specified cancers, Table 3 shows breast cancer was the most common (20.1%), followed by genitourinary cancer (12.7%).

### 3.3. Narrative Summary

**Figure 2:**
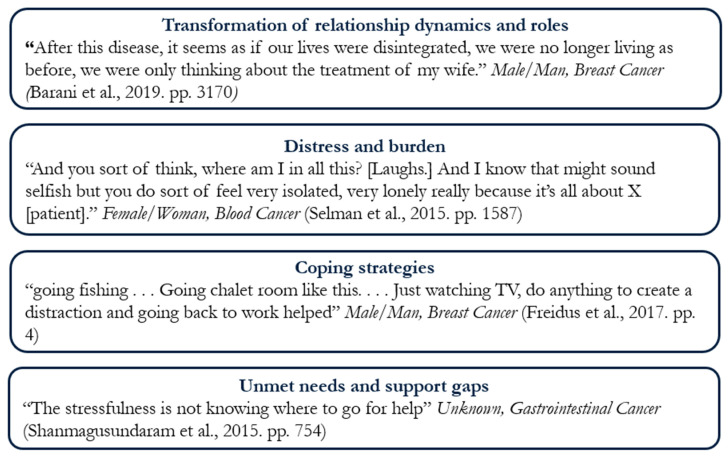
Themes with a representative quote from included studies [27,28,29,30].

Drawing on themes reported across the included studies, we synthesized four themes that captured the lived experiences of partners of patients with cancer: (1) transformation of relationship dynamics and roles, (2) distress and burden, (3) coping strategies, and (4) unmet needs and support gaps. Collectively, these synthesized themes captured the multifaceted nature of partners’ experiences in the context of cancer, highlighting how they adapt to emotional, relational, and practical changes across the illness trajectory (e.g., Figure 2).

### 3.4. Transformation of Relationship Dynamics and Roles

Across most studies, partners became caregivers by shifting their focus from nurturing the relationship to the patient’s recovery while taking on practical and complex medical responsibilities [16,27,31,32]. This responsibility was considered a moral duty or an obligation by partners [14,33,34,35,36,37]. Partners provided support in treatment decision-making while also attempting to respect the patient’s autonomy regarding the partner’s involvement. This was challenging for some partners. For instance, when patients with a brain tumour experienced cognitive decline, partners were described as struggling to respect the patient’s autonomy in decision-making while also trying to provide support [32,38]. The type and stage of cancer determined the magnitude of the caregiving role across studies. For cancer types that required home care, partners indicated they had more tasks along with their routine tasks. Partners provided support with day-to-day hygiene, mobility, medical care (e.g., wound dressing after a surgical treatment), nutrition and specialized feeding for head and neck and esophageal cancers, gathering healthcare-related supplies, and symptom monitoring [31,32,39,40,41,42,43,44,45]. Besides providing day-to-day care, partners also supported scheduling and attending healthcare appointments, tracking medication usage, and advocating for the patient [31]. The intensity of the caregiver role also reportedly increased with progression or metastasis [46]. The support received from healthcare providers was commonly described in studies as inadequate in preparing a partner for this role [31,39,40,47]. The rapid assumption of responsibilities among partners resulted in leisure or personal time being reallocated to the tasks mentioned above, sometimes resulting in reduced work hours or increased time off [48,49,50,51,52].

In addition to the partners’ changed identity of a caretaker from a partner, the relationships also shifted, for example, with changes in emotional connection and sexual intimacy. These changes were attributed to physical limitations, lack of communication, treatment side effects, lack of desire, and the presence of equipment such as feeding tubes and ostomy bags [40,47]. Breast cancer and prostate cancer patients were reported to face challenges with sexual dysfunction and lack of libido, resulting in lower or no intimacy for the partner [43,44,53,54,55,56]. These changes had a continued impact on the relationships even after cancer treatment had ended. Partners reported both positive and negative effects on their relationship as well as their individual self, requiring re-orientation to a post-cancer life [55,57,58,59].

### 3.5. Distress and Burden

With less time for oneself due to caregiving, partners reportedly experienced increased distress and burden that compromised their emotional, physical, and financial wellbeing [15,48,50,60]. Some partners also experienced complex, anticipatory grief and helplessness for the patient in instances of uncertain prognosis associated with metastatic tumours and high-grade gliomas [15,45,49,61,62]. Anxiety around scans and persistent intrusive thoughts were also commonly noted manifestations of distress [15,32,46]. Hospitalizations demanded logistical planning and physical presence [63], which increased stress among partners and required greater time and emotional commitment [64,65]. Several studies noted that hospitalizations and overnight symptom monitoring disrupted the caregivers’ sleep, increased their fatigue, and exacerbated musculoskeletal pain associated with care [52,66,67]. When discharged, partners were concerned about the possibility of making errors, for example, when giving injections, and providing care for drains and ostomies [32,39,40]. Partners’ participation at work was reported to be disrupted due to hospitalizations [50].

This theme is closely linked to the previous, as the primary source of distress and burden stemmed from the caregiving role. For head and neck cancer, partners reported experiencing social stigma and shame, from the limitations imposed by cancer, while handling manual caregiving strain from routine feeding and nutrition [41,47,51,68]. Partners with gastrointestinal and esophageal cancer patients also noted strain from consistent caregiving, with worry about the patient’s food intake, meal planning, and weight [57,69]. Partners of people with prostate cancer were commonly described as experiencing increased loneliness and challenged intimacy [43,70,71]. Studies report that breast cancer patients’ partners were involved in decision-making about breast reconstruction and were distressed about changes in sexuality and shifting couples’ roles [44,54,72]. In studies with prostate, breast, and gynecological cancer patients, partners were also worried about infertility and sexual dysfunction [29,59,73,74]. Studies with hematological cancer patients, partners reported to be physically exhausted from vigilance, repeated admissions, and treatments [39,75,76].

Studies commonly reported that partners often felt overwhelmed [77,78]. They had difficulty concentrating at work [15,67]. Additionally, they faced reduced income sources, increased out-of-pocket expenses (e.g., home supplies, lodging, medications), and greater financial strain on the patient and the partner [51,63,66,79]. Younger partners experienced greater financial distress and overall distress [59,80]. Care in rural areas reportedly demanded greater resources as travel was necessary to access care [63,79]. Partners also experienced shrinking social networks as they had less leisure time and greater fatigue, often leading to increased isolation and loneliness [41,81,82,83]. Emotional isolation was commonly observed among partners due to the demands of their role [84]. Across studies, partners who juggled parenting and employment prioritized practical tasks over emotional support, whereas partners without such responsibilities were more engaged in emotional support [42,82,84]. After the treatment, a fear of recurrence lingered. This fear was more pronounced among younger partners than among older partners [16,57,62].

### 3.6. Coping Strategies

Findings across studies suggested that coping for partners was an act of self and collective preservation. Partners relied on problem-focused, emotion-focused, or meaning-making coping strategies. Such strategies also shifted based on the stage of diagnosis and treatment. Problem-focused coping reportedly included seeking information about cancer and treatment options, clinical skills for caretaking, medication information, and a change in work [39,85]. Emotion-focused coping relied on faith and spiritual practice, distractions, and emotional suppression [15,37,86]. Lastly, studies indicated that meaning-making coping relied on role negotiations, shifts in values, and post-traumatic growth [57,87,88].

The sex of the partner was commonly noted to influence coping approaches. When describing their vulnerabilities, one study found that male partners addressed them as individual vulnerabilities (“me”), whereas female partners addressed them as shared vulnerabilities (“us”) [89]. Further, several studies reported that male partners focused on task-centred coping and engaged in doing over feeling while avoiding emotional support [59,88,90,91]. In contrast, female partners tended to provide more emotional support and to turn to social support networks [35,82,91]. Male cancer patients also tended to withhold communication and suppress emotions, which led to female partners reportedly experiencing more emotional distance and relationship strain [43,92,93]. As a successful coping strategy, couples who grew closer through caregiving were expected to have had more open communication [15,33,94]. Many partners were also described as suppressing their own needs or concerns (including financial ones), as a coping mechanism, to protect the patient and families [15,51,70,71,95,96,97].

Social support systems were considered essential for coping across studies. Informal networks, including family and friends, provided spiritual, practical, and emotional support [79,98]. The presence of peer navigators/caregivers and tumour-specific support groups reportedly helped reduce isolation [14,47,99,100]. With resilience developed from previous hardships and losses, older partners drew strength from experiences of past adversity and spiritual and cultural values [101,102]. Numerous studies reported that partners felt supported when they received clear and compassionate communication from healthcare providers [45,103,104,105,106,107].

### 3.7. Unmet Needs and System Gaps

Attributed to suppression of emotions and lack of time to care for themselves, partners were described as struggling with unmet needs and inadequate support from the healthcare system, which exacerbated their stress and relationship strain [28,32,72,108]. The care provided often did not account for cultural, gender, and life-stage experiences, leaving out key pieces of information for the partner to navigate [30,33,73,109]. With insufficient communication from providers, partners were reported to have received generic information that was overlooked when the couple was overwhelmed [72,78,110]. A subsequent lack of or unorganized follow-ups from providers was commonly noted [61,111]. Due to fear of error, partners wanted more hands-on training for clinical care at home [40,99,111].

As partners had limited authority on deciding treatment options, they often felt sidelined during appointments and expressed a desire for private conversations to learn more details about the patient’s illness [36,93,112]. Being excluded from discussions and decisions reportedly led to increased anticipatory grief and fear of recurrence [16,99]. In addition to these stressors, studies suggested that fragmentation of care also complicated partners’ experiences as they had to navigate the healthcare system [111,113].

Across studies, support for partners was generally absent within the healthcare system [37,49,60,77,114,115,116,117,118,119,120]. The support offered was reported to be centred on the patient, while the partner was left out [121], contributing to caregivers delaying their own care and needs [59,113,121,122]. Psychosocial support was needed, but not without barriers of finances, time, accessibility, etc. [30,41,45,123,124]. The lack of financial support negatively affected younger and lower-income couples. Numerous studies indicated that such financial strain and workplace inflexibility compounded participants’ stress [14,50,51,65,66]. Several studies noted that preparation for end-of-life was severely under-addressed, leaving the experience to be navigated by the partner while caretaking [65,125,126].

## 4. Discussion

This scoping review maps the qualitative research and provides a comprehensive synthesis describing the experiences of partners of individuals diagnosed with cancer. The combined sample of 3042 partners was predominantly middle-aged and with a higher proportion of females (59.2%). By examining 159 included studies, we identified both methodological characteristics of the existing literature and synthesized themes that captured partners’ lived experiences. Drawing on findings across the included studies, being the partners of an individual diagnosed with cancer emerged as an experience marked by shifting relationship dynamics and roles, significant distress and caregiver burden, the use of various coping strategies, and persistent unmet needs and support gaps. These synthesized findings highlight the multifaceted and often interrelated nature of partners’ experiences, revealing how individuals adapt to emotional, relational, and practical challenges throughout the cancer trajectory.

The profound impact of cancer on partners’ lives underscored the reciprocal nature of their experiences with the patient, where each partner’s wellbeing was closely intertwined with that of the person with cancer. Across the included studies, partners’ narratives reflected an interconnection of the four synthesized themes. Following a cancer diagnosis, many partners described a transition in their relationship dynamics as they inadvertently assumed caregiving responsibilities [16,31,32]. While patients were undergoing treatment, partners became the primary source of both practical and emotional support [16,31,32]. Despite their significant influence on the patient’s prognosis and overall wellbeing, partners’ contributions often went unrecognized and insufficiently addressed by healthcare systems [121]. Partners frequently reported feeling isolated, lonely, and fatigued from their caregiving responsibilities [52,66,67,77,78]. With limited time for themselves, their own wellbeing was often neglected. Some partners described attempts to cope by seeking information, maintaining optimism, or drawing on social and spiritual resources [86,101,102]. However, many emphasized the need for improved psychosocial support and clearer communication with healthcare providers to feel adequately prepared for the caregiving role [127].

These support needs were not uniform but varied across cancer types—for example, partners of patients with head and neck cancer sought greater clinical training to feel confident in providing home care, whereas partners of those with prostate, breast, or gynecological cancers wanted opportunities to discuss fertility and sexual health concerns with their providers [40,43,44,75,99,111]. Partners’ experiences were also shaped by individual characteristics, such as sex, and by contextual factors, particularly the type of cancer their partners were diagnosed with. Sex differences were evident across studies, with female partners more frequently describing emotional distress, guilt, and self-neglect from balancing caregiving and household responsibilities [35,82,91]. In contrast, male partners tended to emphasize providing practical or logistical support and often struggled to express or process their emotions [59,86,89,90]. These findings reflect broader patterns of gendered caregiving, where women experience higher levels of psychological strain, and men may face emotional restraint in caregiving roles. The type of cancer also influenced partners’ experiences, as different diagnoses brought unique challenges and care demands [43,55,70,75,83,128].

Strengths and limitations of our scoping review warrant discussion. To our knowledge, this is the first qualitative review particularly centred on the lived experiences of only partners of patients with cancer. Accordingly, most included studies involved samples of partners of patients with cancer or mixed caregiver samples in which partners represented at least half of the participants, meaning that partners made up the majority of the participants rather than other family caregivers or kin. The inclusion of studies representing a wide range of cancer types strengthened our findings, enabling us to identify patterns and variations in partners’ experiences that may be specific to particular cancer trajectories while also highlighting shared challenges across diagnoses. However, including only English-language studies is a limitation, as qualitative data interpretation across languages can introduce challenges in meaning and cultural nuance. While studies from any geographic region were eligible for inclusion, most were conducted in Western countries, suggesting that partners’ experiences in non-Western cultural contexts may be underrepresented. Due to the extent of the studies, it was challenging to report specific data, such as financial and employment constraints. We reported some of these trends in our findings, but it would be useful to explore these in a synthesis of their own, as different factors (e.g., age, socioeconomic status, insurance, work culture, etc.) can influence the intensity of such constraints. Lastly, the construct of “partner” is both complex and dynamic. Our search strategy captured several overlapping terms to reflect this experience; however, inconsistent terminology across studies may have introduced ambiguity, particularly in distinguishing partners from other caregiver roles.

Although we synthesized a large body of qualitative studies, important gaps remain in understanding the full scope and diversity of partners’ experiences in the context of cancer. Future research should prioritize partners whose perspectives have been underrepresented in the literature. Our findings should be generalized with caution; the literature presented in our review underrepresents experiences of more or less marginalized identities facing systemic barriers due to their positionalities. Non-Western, low-income, queer, and young adult partners, among others, can provide valuable insights that broaden and deepen understanding of how social, cultural, and relational contexts shape partners’ experiences. Similarly, examining the experiences of partners of patients who separate during or after treatment may illuminate pathways to support and sustain relationships under the strain of illness.

Overall, this scoping review brings to light the central yet often overlooked role of partners in the cancer experience. By synthesizing qualitative evidence across diverse contexts, it underscores how partners’ emotional, relational, and practical challenges are deeply intertwined with those of the patients they support. Our findings have implications for informing recommendations for both direct and indirect supports for partners of patients with cancer across the cancer pathway. Directly, targeted psychosocial and navigation supports for partners, delivered through patient organizations and aligned with the phase of illness and caregiving intensity, may help address both practical and emotional partner needs. Indirectly, within the health and/or cancer care system, partners’ attendance at appointments may offer opportunities to acknowledge changing caregiving demands at key transition points, particularly across cancer types and illness trajectories, in ways that ultimately support patient care. Clearer communication practices regarding partner roles may further help align expectations around decision-making and caregiving responsibilities. Lastly, recognizing the complexity of these experiences is essential for advancing both research and practice aimed at promoting the wellbeing of individuals living with and alongside cancer [129].

## Figures and Tables

**Table 1:** Characteristics of included studies (*n* = 159).

**Year of Publication**	**No. of Studies (%)**
1986–1990	2 (1.3%)
1991–1995	1 (0.6%)
1996–2000	1 (0.6%)
2001–2005	5 (3.1%)
2006–2010	19 (11.9%)
2011–2015	40 (25.2%)
2016–2020	32 (20.1%)
2021–2025	59 (37.1%)
**Place of Publication**	**No. of Studies (%)**
Europe	49 (30.8%)
North America	44 (27.7%)
Australia	36 (22.6%)
Asia	19 (11.9%)
Africa	10 (6.3%)
South America	1 (0.6%)
**Terminology Used**	**No. of Studies (%)**
Caregiver/Carer	75 (47.2%)
Spouse/Partner/Husband/Wife	71 (44.7%)
Family/Family Members	8 (5%)
Other (e.g., Next of Kin)	4 (2.5%)
Loved One	1 (0.6%)
**Methodology ^1^**	**No. of Studies (%)**
Thematic Analysis	43 (27%)
Content Analysis	32 (20.1%)
Grounded Theory	24 (15.1%)
Mixed	22 (13.8%)
Phenomenological Approaches	21 (13.2%)
Other	10 (6.3%)
Narrative-/Framework-based	4 (2.5%)
Interpretive Description	3 (1.9%)
**Qualitative Data Collection Method Used**	**No. of Studies (%)**
Interviews	147 (92.5%)
Focus Groups	8 (5%)
Focus Groups and Interviews	4 (2.5%)

^1^ Included studies conflated methodology and data analysis methods.

**Table 2:** Characteristics of participants (*n* = 3042) from included studies (*n* = 159).

Participant Characteristics	
**Total Participants**	**3042**
**Sex** ^1^	**No. of Participants (%)**
Female	1780 (59.2%)
Male	1229 (40.8%)
**Age**	**Age (yrs)**
Mean Age	55.2 ± 8.3 years ^2^
Age Range	18–80+ ^3^

^1^ Sex was derived from 156 (*n* = 3009) studies that reported the distribution of sex. ^2^ The mean age was calculated based on 87 studies that reported their mean age. ^3^ The age range was determined based on 133 studies that reported an age range.

**Table 3:** Types of cancer from the included studies.

Cancer Type	No. of Participants (%)
Unspecified/Other	715 (23.5%)
Breast	613 (20.1%)
Genitourinary (Including Prostate)	386 (12.7%)
Gastrointestinal (Including Colorectal)	285 (9.4%)
Neuro-oncology (Central Nervous System)	246 (8.1%)
Thoracic	207 (6.8%)
Head and Neck	212 (7%)
Gynecological	184 (6%)
Hematologic/Immune	138 (4.5%)
Neuroendocrine Tumour (NET)	24 (0.8%)
Skin	24 (0.8%)
Sarcoma/Bone and Soft Tissue	8 (0.3%)

## Data Availability

Data sharing is not applicable to this article as no datasets were generated or analysed for this study.

## Supplementary Materials

The following supporting information can be downloaded at https://www.mdpi.com/article/10.3390/curroncol33020069/s1. Table S1: Embase Ovid search (1974 to 19 February 2025). Table S2: Ovid MEDLINE(R) (1946 to 19 February 2025). Table S3: CINAHL (1974 to 19 February 2025). Table S4: PsycInfo (1974 to 19 February 2025). Table S5: Scopus (1974 to 19 February 2025). Table S6: List of included studies.
