# Supplementary material for: “The Day He Fell Ill, We Turned on a Switch…Now, Everything Is My Responsibility”: Scoping Review of Qualitative Studies Among Partners of Patients with Cancer
Source: Curr Oncol. 2026 Jan 24;33(2):69. doi: 10.3390/curroncol33020069 (PMC12939333; doi:10.3390/curroncol33020069)
Supplement: Supplementary file 1 [file curroncol-33-00069-s001.zip › curroncol-4049276-SI.pdf]

SUPPLEMENTARY MATERIAL

Qualitative search terms were adapted in part from University of Pittsburgh, Ovid MEDLINE Search Filters: Qualitative Studies (https://hsls.libguides.com/Ovid-Medline-search-filters/qualitative-studies)

Table S1. Embase Ovid Search (1974 to February 19, 2025)

Search line	Search	Hits
1	(partner? or husband* or wife or wives or boyfriend* or girlfriend* or spous* or marital or marriage* or common law or significant other* or romantic or couple* or intimate or same sex).ti,ab,kf.	915958
2	caregiv*.ti,ab,kf.	154469
3	Spouses/	21092
4	Marriage/	80642
5	or/1-4	1085774
6	exp Neoplasms/	6185626
7	(cancer* or neoplasm* or oncolog*).ti,ab,kf.	3907936
8	6 or 7	6833867
9	document analysis/ or exp interview/ or qualitative research/ or ((depth or face or group or guided or indepth or informal or semistructured or structured or unstructured) adj4 (discussion or discussions or interview or interviewed or interviews or questionnaire or questionnaires)).ti,ab,kf,kw. or (ethnographic or ethnography or (field adj1 work) or fieldwork or (focus adj1 (group or groups)) or (groups adj2 interviewed) or (key adj1 (informant or informants)) or (qualitative adj2 (research or studies or studies or synthesis))).ti,ab,kf,kw.	706371
10	(phenomenol* or (grounded adj (theor* or study or studies or research or analys?s)) or lived experience* or (thematic adj2 analys?s)).ti,ab,kf.	143481
11	qualitative.ti,kf.	126862
12	9 or 10 or 11	779237
13	5 and 8 and 12	9916
14	limit 13 to (english language and embase)	4085

Table S2. Ovid MEDLINE(R) and Epub Ahead of Print, In-Process, In-Data-Review & Other Non-Indexed Citations, Daily and Versions(R) Search <1946 to February 19, 2025>

Search line	Search	Hits
1	(partner? or husband* or wife or wives or boyfriend* or girlfriend* or spous* or marital or marriage* or common law or significant other* or romantic or couple* or intimate or same sex).ti,ab,kf.	775019
2	caregiv*.ti,ab,kf.	114504
3	Spouses/	12199
4	Marriage/	25536
5	Sexual Partners/	21457
6	or/1-5	893318
7	exp Neoplasms/	4075295
8	(cancer* or neoplasm* or oncolog*).ti,ab,kf.	2833483
9	7 or 8	4901283
10	Document Analysis/ or focus groups/ or interviews as topic/ or narration/ or qualitative research/ or ((depth or face or group or guided or indepth or informal or semistructured or structured or unstructured) adj4 (discussion or discussions or interview or interviewed or interviews or questionnaire or questionnaires)).ti,ab,kf,kw. or (ethnographic or ethnography or (field adj1 work) or fieldwork or (focus adj1 (group or groups)) or (groups adj2 interviewed) or (key adj1 (informant or informants)) or (qualitative adj2 (research or study or studies or synthesis))).ti,ab,kf,kw.	430441
11	(phenomenol* or (grounded adj (theor* or study or studies or research or analys?s)) or lived experience* or (thematic adj2 analys?s)).ti,ab,kf.	123842
12	qualitative.ti,kf.	114178
13	10 or 11 or 12	496380
14	6 and 9 and 13	4917
15	limit 14 to english language	4822

Table S3. CINAHL (1974 to February 19, 2025)

Search line	Search	Hits
S1	(partner# or husband* or wife or wives or boyfriend* or girlfriend* or spous* or marital or marriage* or common law or significant other* or romantic or couple* or intimate or same sex or caregiv*)	276,538
S2	(MH "Spouses")	13,743
S3	(MH "Marriage+")	10,097
S4	(MH "Sexual Partners")	10,709
S5	S1 OR S2 OR S3 OR S4	276,538
S6	(MH "Neoplasms+")	673,190
S7	cancer* or neoplasm* or oncolog*	810,212
S8	S6 OR S7	892,718
S9	(MH "Qualitative Studies+")	203,979
S10	(MH "Interviews+") OR (MH "Focus Groups")	306,180
S11	(depth or face or group or guided or indepth or informal or semistructured or structured or unstructured) N4 (discussion* or interview* or questionnaire*)	208,252
S12	ethnograph* or (field N1 work) or fieldwork or (focus N1 group*) or (groups N2 interviewed)	87,033
S13	(key N1 informant*) or phenomenol* or (grounded N1 (theor* or study or studies or research or analys?s)) or "lived experience*" or (thematic N2 analys?s))	163,379
S14	TI qualitative	56,753
S15	S9 OR S10 OR S11 OR S12 OR S13 OR S14	480,850
S16	S5 AND S8 AND S15	5,086
S17	S5 AND S8 AND S15 [limited to English and academic journals]	4,614

Table S4. PsycInfo (1974 to February 19, 2025)

Search line	Search	Hits
S1	(partner# or husband* or wife or wives or boyfriend* or girlfriend* or spous* or marital or marriage* or common law or significant other* or romantic or couple* or intimate or same sex or caregiv*)	397,712
S2	DE "Couples" OR DE "Significant Others" OR DE "Spouses" OR DE "Partners" OR DE "Sexual Partners" OR DE "Marriage" OR DE "Same Sex Marriage"	63,220
S3	S1 OR S2	397,712
S4	DE "Neoplasms" OR DE "Breast Neoplasms" OR DE "Digestive System Neoplasms" OR DE "Endocrine Neoplasms" OR DE "Leukemias" OR DE "Lung Neoplasms" OR DE "Metastasis" OR DE "Nervous System Neoplasms" OR DE "Skin Neoplasms" OR DE "Terminal Cancer"	66,210
S5	cancer* or neoplasm* or oncolog*	118,026
S6	DE "Qualitative Methods" OR DE "Coding Scheme" OR DE "Content Analysis" OR DE "Ethnography" OR DE "Focus Group" OR DE "Grounded Theory" OR DE "Interpretative Phenomenological Analysis" OR DE "Narrative Analysis" OR DE "Semi-Structured Interview" OR DE "Thematic Analysis"	41,128
S7	(depth or face or group or guided or indepth or informal or semistructured or structured or unstructured) N4 (discussion* or interview* or questionnaire*)	249,686
S8	ethnograph* or (field N1 work) or fieldwork or (focus N1 group*) or (groups N2 interviewed)	98,648
S9	(key N1 informant*) or phenomenol* or (grounded N1 (theor* or study or studies or research or analys?s)) or "lived experience*" or (thematic N2 analys?s))	142,800
S10	TI qualitative	43,141
S11	S7 OR S8 OR S9 OR S10 OR S11	425,976
S12	S3 AND S6 AND S12	2,377
S13	S3 AND S6 AND S12 [limited to English and academic journals]	2,096

Table S5. Scopus (1974 to February 19, 2025)

Search	Hits
(TITLE-ABS-KEY (partner? OR husband* OR wife OR wives OR boyfriend* OR girlfriend* OR spous* OR marital OR marriage* OR common AND law OR significant AND other* OR romantic OR couple* OR intimate OR same AND sex OR caregiv*)) AND (TITLE-ABS-KEY (cancer* OR neoplasm* OR oncolog*)) AND ((TITLE-ABS-KEY ((depth OR face OR group OR guided OR indepth OR informal OR semistructured OR structured OR unstructured) W/4 (discussion* OR interview* OR questionnaire*))) OR (TITLE-ABS-KEY (ethnograph* OR (field W/1 work) OR fieldwork OR (focus W/1 group*) OR (groups W/2 interviewed))) OR (TITLE-ABS-KEY ((key W/1 informant*) OR phenomenol* OR (grounded W/1 (theor* OR study OR studies OR research OR analys?s)) OR "lived experience*" OR (thematic W/2 analys?s))) OR (TITLE-ABS-KEY (qualitative))) AND (LIMIT-TO (LANGUAGE , "English"))	112

Table S6. List of Included Studies

No.	Author (last name)	Year	Country	Aim
1	Acquati	2023	USA	To identify the challenges partners of BCS encounter, the strategies used to cope with these challenges, and the recommendations they have for providers.
2	Anderson	2019	Denmark	To investigate needs of caregivers for individuals with cancer and to gain insight into how to provide support for caregivers
3	Angelo	2014	New Zealand	To explore occupational roles of hospice family caregivers among Māori, Chinese, and Tongan ethnic groups in New Zealand with respect to caring for dying family members
4	Anngela-Cole	2011	USA	To explore informal carers' in different cultures respond to their in caregiving role
5	Anthias	2016	UK	To understand partner perspective in late-stage cancer
6	Anu	2022	Finland	To explore psychosocial support in palliative care
7	Appiah	2024	Ghana	To explore experiences of spouses of men with prostate cancer in Ghana
8	Aranda	2001	Australia	To understand lay decision making for home palliative caregiving in advanced cancer
9	Arber	2013	UK	To explore support needs for carers of malignant brain tumour
10	Bahrami	2024	Iran	To explore the experiences of family caregivers of patients withadvanced cancer regarding the hidden emotional concerns associated with caregiving for loved ones.
11	Balfe	2016	Ireland/UK	To explore financial impact of head and neck cancer caregiving
12	Bamidele	2019	UK	To explore experience of partners of Black African/Caribbean men with prostate cancer
13	Banks	2023	Australia	To explore fear of cancer recurrence in caregivers
14	Barani	2019	Iran	To understand experiences of spouses of breast cancer patients
15	Benkel	2012	Sweden	To examine how and what kind of information the loved ones obtained about the disease, which supported their understanding, realization, and preparation of the patient’s approaching death.

16	Boamah Mensah	2021	Ghana	To explore experiences, social stressors & resources husbands of women with advanced breast cancer rely on (primary caregivers)
17	Bostrom	2003	Norway	To illuminate the meaning of being a female or male partner of a young patient who had been diagnosed and survived the cancer experience
18	Broback	2023	Sweden	To identify and describe the next of kin's experiences of palliative care in the home.
19	Bruun	2011	Denmark	To understand life with incurable prostate cancer from the perspective of the female partner, with the intent of better understanding female partners' everyday experiences.
20	Carter	2001	USA	To highlight the need for holistic health care practitioners to evaluate their caregivers' individual needs for information and to attempt to provide this information in a sensitive yet factually accurate manner.
21	Catania	2019	Malta	To investigate the experiences of male partners of female breast cancer patients who had undergone surgery and oncological treatment and who were still raising children.
22	Cheng	2014	Canada	To assess the perceived needs and preferred services of male partners of women newly diagnosed with breast cancer.
23	Cheng	2022	Canada	To explore male partners' coping style of self-reliance, and the perceived social support that these partners relied upon to cope with their wife's breast cancer
24	Clemmer	2008	Canada	To explore the roles experienced by family members providing home-based palliative care to seniors with advanced cancer.
25	Coenen	2023	Netherlands	To explore experienced health- and work-related problems of partners of patients with cancer, and their needs for support.
26	Coolbrandt	2015	Belgium	To explore the experience of family caregivers of patients with HGG and their needs related to professional care.
27	Coppetti	2025	Brazil	To understand the meaning attributed by men to the experience of caring for their family member with cancer and develop a substantive theory that represents the experience of men in caring for their family member with cancer.
28	Cormican	2023	Ireland	To explore carers' experiences, expressed needs and psychosocial vulnerability of haematological cancers

29	Dalhberg	2025	Sweden	To explore how informal caregivers of persons living with brain tumors use resources available to them, including social support, to balance caregiving with other aspects of life.
30	Does	1989	Netherlands	To explore experiences of men during the period their wives were hospitalized for cervix cancer and during recovery
31	Dri	2020	Italy	To explore the lived experience of primary family caregivers of HNC patients dealing with laryngectomy regarding their complex supportive role.
32	Duggleby	2012	Canada	To explore the hope experiences of male spouses of women with breast cancer
33	Egestad	2019	Denmark	To explore the experiences of the partners of men with breast cancer in relation to care, information, and emotional support and to explore how men with breast cancer impact the partners' everyday life.
34	Ervik	2013	Norway	To explore how the daily life of female spouses is affected by their husband's prostate cancer.
35	Esbensen	2010	Denmark	To illuminate the experience of next of kin of elderly people with cancer.
36	Evertsen	2010	USA	To explore women's interactions with physicians (primary care and urologist) and the support needs associated with the diagnosis and treatment of their partners' prostate cance
37	Fitch	2007	Canada	To explore the impact on male partners of having a wife who has been diagnosed with breast cancer.
38	Francis	2020	Denmark	To explore ethical dilemmas spouses experience in everyday care of a partner with brain tumour.
39	Francis	2022	Denmark	To investigate spouses' experiences of suffering in caregiving role.
40	Freidus	2017	US	To explore men's experiences committing to relationships with younger breast cancer survivors.
41	Funk	2009	Canada	To explore family caregiver accounts of healthcare experiences, focusing on concept of "security."
42	Gao	2020	China	To investigate supportive strategies of husbands of women with breast cancer across diagnosis, treatment, survivorship.
43	Gerhardt	2020	Denmark	To explore experiences of caregivers during follow-up after curative treatment for pancreatic, duodenal, bile duct cancers.
44	Germeni	2015	Greece	To understand experiences of socioeconomically deprived caregivers in Greece, esp. barriers to home-based cancer care.
45	Gilbert	2008	Australia	To explore how carers renegotiate sexuality and intimacy in the context of cancer

46	Gilbert	2009	Australia	To examine disruptions to sexuality for partners caring for a person with cancer, and how caring role/social norms shape experiences.
47	Gosse	2024	Tanzania	To explore experiences of male partners caring for women with cervical cancer in Dar es Salaam
48	Goswami	2019	India	To understand psychosocial impact and coping mechanisms of family caregivers of oral cancer patients
49	Gunn	2022	Australia	To explore rural Australian carers' experiences and impact of caregiving on well-being
50	Gupta	2024	US	To characterize sexual health concerns and unmet needs of female partners of prostate cancer survivors
51	Gutierrez	2016	US	To explore the influence of breast cancer on Latino male partners of Latina survivors, and provide insight into their coping, support strategies, and cultural context (machismo/caballerismo).
52	Halkett	2020	Australia	To explore lived experiences of carers of patients diagnosed with head and neck cancer
53	Hansen	2017	US	To explore family caregivers' perspectives of caring for patients with terminal hepatocellular carcinoma
54	Harrison	2021	Australia	To explore caregiver roles in clinical care provision and their support needs, especially in priority populations
55	Hashemi-Ghasemabadi	2016	Iran	To describe transition to caregiving role among Iranian family caregivers of breast cancer patients
56	Heckel	2018	Germany	To compare experiences and needs of informal caregivers of brain tumor vs. non-brain tumor patients in home care settings
57	Hiatt	2022	Australia	To understand and compare nutrition care experiences of carers supporting HNC patients through surgery and radiation
58	Hilton	2000	Canada	To describe men's experiences and coping as partners, fathers, and caregivers during wives' breast cancer and chemotherapy
59	Huang	2019	Taiwan	To explore lived experience of spousal caregivers of newly diagnosed cancer patients in Taiwan
60	Iannarino	2018	US	To explore biographical disruption and communication work among close supporters of young adult cancer survivors
61	Ibrahim	2020	Sweden	To explore next-of-kin experiences of participation in surgical cancer care for upper GI malignancies
62	Jepsen	2019	Denmark	To explore how outpatient management affects the everyday life and psychosocial well-being of spouses to AL patients

63	Johnston	2024	Australia	To investigate rural caregivers' experiences seeking support for their own health and wellbeing while caring for someone with cancer
64	Johnston	2024	Australia	To explore how caring for someone with cancer affects rural caregivers' health behaviors and identify socioecological factors underlying these changes
65	Agbodjavou	2024	Togo	To explore factors that reinforce feelings of safety and insecurity among family caregivers providing end-of-life care at home for relatives with diabetes and/or advanced cancer in Togo.
66	Jaurez	2014	USA	To describe the caregiving experience and challenges of caregivers of Mexican ancestry, emphasizing cultural values, commitment, daily life challenges, and spirituality.
67	Jaurez	2015	USA	To explore how culture influences perceptions of quality of life (QOL) among caregivers of Mexican ancestry caring for adults with advanced cancer, across physical, psychological, social, and spiritual domains.
68	Ka'opua	2025	USA	To describe the adaptive challenges and coping strategies of elderly Asian/Pacific Islander wives of long-term prostate cancer survivors, with attention to cultural and ethnic influences.
69	Kenny	2020	Australia	To explore Australian male caregivers' experiences of informal cancer care, focusing on tensions around masculinity, reciprocity, and vulnerability.
70	Kenny	2023	Australia	To explore informal caregivers' perspectives on precision medicine in cancer care, especially the relational and emotional dimensions of hope.
71	Kidd	2011	Germany	To determine the supportive care needs of unpaid carers within British Forces Germany caring for someone with cancer.
72	King	2024	US	To explore perceptions of loneliness, spirituality, and their relationship to health-related quality of life among Hispanic cancer caregivers.
73	Kirby	2022	Australia	To explore the lived experience of informal caregivers of people with glioma, focusing on social isolation, emotional burden, and relational dynamics.
74	Komariyah	2024	Indonesia	To explore husbands' perceptions and experiences while caring for wives with cervical cancer, including emotional, sexual, and support-related dimensions.
75	Lally	2025	USA	To explore the experiences, roles, decision-making, and coping strategies of care partners co-surviving with women living with metastatic breast cancer.

76	Langegard	2023	Sweden	to explore informal caregivers' views on their challenges and needs in attaining high preparedness for<br>caregiving.
77	Larsen	2021	Denmark	To explore relatives' experiences with illness, treatment, and decision-making in the context of esophageal cancer.
78	Leonidou	2018	Cyprus	To examine metastatic cancer caregivers' experiences, unmet needs, and support resources in a Cypriot sample.
79	Lewis	2020	Australia	To examine caregivers' experiences of cancer prognosis and their role in prognostic conversations.
80	Liang	2019	Taiwan	To describe primary caregiving tasks and challenges faced by family caregivers of oral cancer patients in Taiwan.
81	Lin	2013	Taiwan	To explore the lived experiences of male spouses caring for wives with metastatic cancer in Taiwan.
82	Lin	2015	Taiwan	To explore the lived experience of middle-aged female spouses caring for husbands with advanced cancer.
83	Lion	2024	Australia	To explore caregivers' experiences of psychological support and perceptions of optimal support in the context of high-grade glioma.
84	Lohfeld	2007	Canada	To examine continuity and discontinuity of care from the perspective of spousal caregivers using Dumont et al.'s theoretical model.
85	Lopez	2012	UK	To explore male spouses'/partners' experience of caring for their wives/partners with breast and gynecologic cancer over a 1-year period.
86	Maleki	2022	Iran	To explore changes and challenges in sexual life experienced by the husbands of women diagnosed with breast cancer.
87	Mbozi	2023	Zambia	To explore experiences, needs and coping strategies of wives caring for husbands with cancer at CDH; identify challenges, facilitators, needs and coping.
88	McConigley	2010	Australia	To articulate experiences and information/support needs of family caregivers of people with high-grade glioma; describe rapidity of change and support needs.
89	McDonald	2018	Canada	To conceptualise caregiver QOL from caregivers' perspective and compare themes between early palliative care vs usual care arms.
90	Melin-Johansson	2007	Sweden	To describe caregivers' perceptions of terminally ill family members' quality of life

91	Milberg	2004	Sweden	To describe and interpret comprehensibility and manageability for informal carers in hospital-based palliative home care
92	Mok	2003	Hong Kong	To explore caregiving process, meaning, and impact for Chinese family caregivers of terminally ill cancer patients
93	Mosher	2013	USA	To identify key challenges experienced by distressed family caregivers of lung cancer patients
94	Mosher	2015	USA	To identify need, enabling, and psychosocial predisposing factors associated with non-use of mental health services among distressed caregivers
95	Msengi	2024	Tanzania	To explore coping mechanisms used by male partners of women diagnosed with cervical cancer
96	Murrell	2023	UK	To explore the nature and quality of support from informal networks for informal caregivers of LGG patients
97	Nasiri	2012	Iran	To explore sexual issues of Iranian men after breast cancer in their wives
98	Neves	2022	Portugal	To understand lived experience of spouses accompanying young adults at end-of-life
99	Ninnoni	2023	Ghana	To explore psychosocial experiences of family caregivers of prostate cancer patients
100	Noveiri	2021	Iran	To explore concept of coping from lived experiences of male spouses of women with breast cancer
101	Noveiri	2022	Iran	To clarify challenges faced by spouses of women with breast cancer using lived experience
102	Olson	2013	Australia	To explain variation in cancer carers' emotional experiences and support preferences using a sociological time-sovereignty framework
103	Olson	2011	Australia	To contribute a sociological account of carers' coping strategies at diagnosis and introduce the concept of temporal anomie
104	Olson	2014	Australia	To explore cancer spouse carers experiences of loss and grief and introduce the concept "indefinite loss"
105	Olson	2012	Australia	To examine Canberra-based spouse carers experiences after shift of care to home; focus on carers role coordinating patient care and support needs
106	Opsomer	2024	Belgium	To identify prototypical longitudinal trajectories of resilience/distress in partners of patients diagnosed with advanced cancer and map resilience-promoting resources

107	Osei	2024	Ghana	To explore multifaceted impacts of cervical cancer on male spousal caregivers and identify coping and support mechanisms
108	Ownsworth	2015	Australia	To investigate caregivers perceptions of their support needs and the impact of brain tumor on relationship functioning
109	Owoo	2022	Ghana	To describe physical impacts of caregiving for prostate cancer patients (sleep, fatigue, pain, nutrition)
110	Owoo	2022	Ghana	To explore physical impact of caring for prostate cancer patients
111	Penrod	2011	USA	To illustrate variations in caregiving trajectories across three distinct death trajectories
112	Pinks	2018	Australia	To deeper understanding of their experiences of prostate cancer survivorship to help inform healthcare service providers.
113	Ponto	2008	USA	To describe experience of ovarian cancer from husbands' perspective
114	Pusa	2011	Sweden	To illuminate lived experiences of significant others from diagnosis through bereavement
115	Rimmer	2024	UK	To explore the emotional impact of caregiving for someone with a lower-grade glioma
116	Roen	2018	norway	To explore how healthcare providers can support resilience in carers of advanced cancer patients
117	Roing	2008	Sweden	To describe oral cancer and its initial treatment as experienced by the patients spouses.
118	Samuelsson	2025	Sweden	To describe variations in family members' supportive care needs across the CRC trajectory
119	Schmer	2008	USA	To explore caregiver experiences during first 6 months of treatment for malignant brain tumor
120	Selman	2015	UK	To describe the impact of CTCL on family members and how they cope and adjust, to inform support services.
121	Shanmugasundaram	2015	Australia	To explore unmet needs of Indian family caregivers receiving palliative care in Australia
122	Shannon	2015	Canada	To explore men's leisure experiences while caregiving for partners during breast cancer
123	Shaw	2013	Australia	To explore caregiver role perception and unmet supportive care needs post-surgery
124	Sherwood	2011	USA	To examine caregiver role transition and changing perceptions over first 4 months
125	Sinclair	2024	Australia	To explore experiences and support needs of partners of women diagnosed with cancer during pregnancy
126	Stajduhar	2008	Canada	To identify factors influencing caregiver coping in home-based end-of-life care

127	Stamataki	2014	UK	To test the Cancer Family Caregiving Experience model
128	Stenberg	2012	Norway	To understand lived experiences of caregivers across cancer types and trajectories
129	Stetz	1987	USA	To describe caregiving demands experienced by spouses of patients with advanced cancer
130	Street	2008	Australia	To explore coping and psychosocial adaptation in female partners of men with prostate cancer
131	Sutherland	2009	UK	To explore the meaning of being in transition to end-of-life care among female partners of spouses with cancer
132	Swanberg	2006	USA	To identify workplace factors that inhibit or facilitate caregivers' ability to provide care and aspects of caregiving that hinder meeting work responsibilities.
133	Tabler	2015	USA	To explore whether and how hospice addresses bereavement-related needs of family caregivers (pre- and post-death) and identify missed opportunities.
134	Tang	2019	China	To understand how caregiving influences FCs' daily lives and to identify challenges, personal growth, and social support needs among FCs of terminally ill cancer patients.
135	Taylor	2021	Australia	To explore sources of distress among diverse caregivers across disease stages including bereavement
136	Taylor	2021	Australia	To explore barriers and preferences for accessing supportive care for caregivers' own wellbeing
137	Traboulssi	2022	Bahrain	To explore lived experience, coping, and support needs of Arab men whose wives had breast cancer
138	Tranberg	2021	Sweden	To explore perceived burden and identity loss among caregivers seeking peer support
139	Trudeau-Hern	2012	USA	To examine biopsychosocial health impact of caregiving on spousal caregivers, including chronic illness risk
140	Ugalde	2012	Australia	To explore how caregivers view their role and the impact of caregiving on self-identity
141	Urbutiene	2025	Lithuania	To explore death-related experiences in spouses of cancer survivors in remission in context of fear of recurrence
142	Ussher	2013	Australia	To examine gendered experiences of difficulties and rewards in cancer caregiving
143	Ussher	2010	Australia	To examine the nature and consequences of cancer on roles and relationship dynamics between people with cancer and their carers

144	Wahid	2024	Malaysia	To explore husbands' perspectives on sexuality and coping with wives' sexual dysfunction after breast cancer
145	Ward-Griffin	2012	Canada	To describe end-of-life care provision from caregivers' perspective and portray dialectical relational care experiences in home-based care
146	Washington	2019	USA	To explore caregivers' preferences and experiences regarding communication with oncology providers
147	Washington	2021	USA	To identify and describe comfort needs of cancer family caregivers receiving outpatient palliative care
148	Waters	2021	USA	To describe YACC experiences, burden, and social support, and implications for tailored services
149	Weaver	2022	Australia	To understand carers' perspectives of the HNC role and perceived expectations, including nutrition responsibility and relationship change
150	Webb	2024	Australia	To explore caregiver experiences of fear of cancer recurrence/progression and implications for caregiver-specific interventions
151	Weiss	2024	USA	To identify I-Poems within caregiver narratives and explore themes of personal becoming/transformation
152	Williams	2014	USA	To describe wives' experiences and strategies during husbands' recovery; develop a framework of normalization processes
153	Winter	2024	USA	To understand caregivers' adaptation, meaning-making, and resilience processes early in HM diagnosis/treatment
154	Wootten	2014	Australia	To explore partners' experiences and personal impact to inform supportive interventions
155	Yoshimochi	2018	Brazil	To understand partners' lived experience and needs while caring for women with breast cancer
156	Young	2022	UK	To explore men's perspectives of caring for a female partner with cancer over one year
157	Yuen	2025	Australia	To examine caregiver-perceived factors that contribute to social connection during cancer caregiving
158	Zahlis	1993	USA	To illuminate reasons male partners continue to feel distress 18 months after their wives' breast cancer diagnosis
159	Zahlis	2010	USA	To describe men's experiences and processes during the first six months of their wives' breast cancer diagnosis and treatment
